# From Extraction of Local Structures of Protein Energy Landscapes to Improved Decoy Selection in Template-Free Protein Structure Prediction

**DOI:** 10.3390/molecules23010216

**Published:** 2018-01-19

**Authors:** Nasrin Akhter, Amarda Shehu

**Affiliations:** 1 Department of Computer Science, George Mason University, Fairfax, VA 22030, USA; nakhter3@gmu.edu; 2 Department of Bioengineering, George Mason University, Fairfax, VA 22030, USA; 3 School of Systems Biology, George Mason University, Mansassas, VA 20110, USA

**Keywords:** template-free protein structure prediction, decoy selection, conformational space, energy landscape, basins, Pareto optimality

## Abstract

Due to the essential role that the three-dimensional conformation of a protein plays in regulating interactions with molecular partners, wet and dry laboratories seek biologically-active conformations of a protein to decode its function. Computational approaches are gaining prominence due to the labor and cost demands of wet laboratory investigations. Template-free methods can now compute thousands of conformations known as decoys, but selecting native conformations from the generated decoys remains challenging. Repeatedly, research has shown that the protein energy functions whose minima are sought in the generation of decoys are unreliable indicators of nativeness. The prevalent approach ignores energy altogether and clusters decoys by conformational similarity. Complementary recent efforts design protein-specific scoring functions or train machine learning models on labeled decoys. In this paper, we show that an informative consideration of energy can be carried out under the energy landscape view. Specifically, we leverage local structures known as basins in the energy landscape probed by a template-free method. We propose and compare various strategies of basin-based decoy selection that we demonstrate are superior to clustering-based strategies. The presented results point to further directions of research for improving decoy selection, including the ability to properly consider the multiplicity of native conformations of proteins.

## 1. Introduction

Protein molecules control virtually all processes that maintain and replicate a living cell. The conformations in which the sequence of amino acids that constitute a protein molecule fold in three-dimensional space are central to its biological activities in the cell. The geometric and physico-chemical complementary of molecular conformations determine events such as binding and docking, making the ability of a protein to assume specific conformations essential to regulation of interactions with molecular partners [1]. Due to the central role that conformations of a protein play in governing recognition events, significant efforts in wet laboratories are devoted to determination of biologically-active conformations as a means of decoding protein function. This task is growing in urgency due to the millions of uncharacterized protein-encoding gene sequences deposited in genomic databases by increasingly faster and less expensive high-throughput gene sequencing technologies [2].

As of December 4th 2017, the Protein Data Bank (PDB: http://www.rcsb.org/pdb) [3] where wet laboratories deposit determined biologically-active conformations contains 125,799 such conformations for 42,841 distinct protein sequences. The disparity between the number of conformations and the number of sequences is due to the high labor and cost demands of wet laboratory methods. As a result, computational methods are gaining prominence. In particular, template-free methods, which focus on obtaining biologically-active conformations of a protein from knowledge of its amino-acid sequence (in absence of a conformational template from a homologous sequence), are improving in their capabilities [4]. These methods, the most popular of which include Rosetta [5] and Quark [6], operate under the umbrella of stochastic optimization and compute conformations by proving local minima of a selected energy/scoring function that sums atomic interactions [7].

Template-free methods face a daunting task for two reasons. First, the conformation space they explore in search of biologically-active conformations is vast and continuous due to the large number of conformations in which a sequence of amino acids can fold. This number grows exponentially with the number of amino acids. Second, it is unclear what makes a conformation biologically-active. This is also known as the nativeness problem. It is now well understood that energy functions designed and optimized to obtain conformations of a protein sequence are unreliable indicators of nativeness; that is, low energy does not correlate with nativeness. For this reason, identifying one or more native conformations from the set of (decoy) conformations computed by a template-free method, a problem known as decoy selection, remains challenging.

The Critical Assessment of protein Structure Prediction (CASP) series of community-wide experiments [8] was introduced two decades ago to advance and assess progress by template-free methods. CASP challenges participants to submit blind predictions of about one hundred (target) proteins. The CASP challenge runs for three months every other year, and submitted conformations are evaluated by independent assessors after one or a few biologically-active conformations per target are made available by wet laboratory investigators. Biennial assessments of CASP, such as the latest one in [9], demonstrate the growing capability of template-free methods to feasibly generate thousands of diverse, low-energy conformations of a protein sequence. This ability helps with decoy selection, in that it provides more data for methods that seek to recognize native conformations among decoys.

The latest CASP assessment [9] shows that decoy selection remains a bottleneck. Repeatedly, research has shown that setting an energy threshold either misses native conformations or allows the inclusion of too many non-native ones. A popular approach for decoy selection for more than a decade has been to ignore energy altogether and organize decoys by their conformational similarity via clustering [8,10,11]. Once clustering has been performed, the *k* highest-populated clusters, with common values of *k* varying from 1 to 10, are typically offered as prediction [12]. This strategy has varied success, and its utility is tightly related to the quality of the generated decoys [12].

The premise in cluster-based decoy selection methods is that decoys are randomly distributed around the true answer, which a consensus-seeking method ought to reveal. There are two major issues with this premise. First, due to the size and dimensionality of the conformation space, the decoy sampling process in template-free methods employs heuristics and biases that do not guarantee that the decoys provide a uniformly-sampled view of the conformation space. In particular, energy functions designed for template-free methods contain in them inherent biases that invalidate entire regions of the conformation space. Second, there is often no single true answer, as proteins are intrinsically-dynamic systems capable of populating distinct conformations with which they bind to different molecules in the cell. Though in CASP, the assessment is with respect to one native conformation determined in the wet laboratory, the multiplicity of native conformations cannot be ignored [13,14,15,16].

Cluster-based methods fail to pick up exceptionally-good decoys and are especially weak when applied to hard targets where decoys are typically highly dissimilar (and sparsely sampled) [8]. For this reason, two growing thrusts of research focus on designing new, statistical scoring functions that can assess the quality of a single decoy [17,18] and machine learning (ML) methods (often in combination with statistical scoring functions) trained on labeled decoys [19]. Though in their infancy, these directions are showing promise. They have to overcome many challenges, including model generalization and transferability; that is, the ability to be applicable to different decoy datasets.

In light of these remaining challenges and the recognition that via decoy sampling, template-free methods probe an underlying energy landscape, we propose and evaluate here a complementary approach to decoy selection. The approach does not ignore energy, but instead takes it into account under the energy landscape view that relates biologically-active conformations to thermodynamic stability (and function) [20,21,22,23,24]. Utilizing recent spatial data analytics techniques, we seek and extract local structures from a sampled energy landscape. Throughout this paper, we elect to use the term protein conformation rather than structure, reserving the term structure to indicate organization in the energy landscape associated with a protein’s conformational space. These structures, referred to as basins, correspond to the stable and semi-stable conformational states (to the extent that such states are sampled by a template-free method) utilized by a protein to carry out a possibly diverse menu of biological activities. Once the decoys are organized into basins, characteristics of basins are then leveraged here to propose various basin selection strategies that are evaluated thoroughly and in detail. in our journal, moved to main text, please confirm.

The proposed selection strategies include ones based on Pareto optimality, which are demonstrated to be superior to cluster-based and other basin-based selection strategies proposed in this paper. While the concept of Pareto optimality is described in detail in Section 4 in this paper, the main idea is how to select items or objects (in our case, basins) that can be described by conflicting characteristics/criteria; that is, addressing selection in cases where focusing on improving one criterion may worsen one or more of the other criteria. Pareto-based selection allows addressing the presence of multiple conflicting criteria. Moreover, inspired by an ML treatment, evaluation metrics are proposed in this paper to demonstrate the presence or not of structure in a sampled energy landscape, thus exposing targets that are trivial or hard for template-free methods, and to rigorously compare decoy selection strategies in a blind prediction setting.

The rest of this paper is organized as follows. First, for the interested reader, we provide a more detailed (but not exhaustive) overview of related work in decoy selection in below. The evaluation of proposed and baseline selection strategies is presented in Section 2, and a discussion follows in Section 3. The proposed strategies are described in detail in Section 4.

### Related Work

Decoy selection literature features a number of works suggesting the utility of energy/scoring functions that fall into two broad categories, statistical versus physics-based. Statistical functions, also referred to as knowledge-based functions, rely on statistical analysis of known native conformations deposited in the PDB, whereas physics-based functions consider the physical principles of atomic interactions. Despite the apparent superiority of statistical functions in discriminating native conformations supported by a large body of work [25,26,27,28,29,30,31], some physics-based functions have also been proved effective in decoy evaluation [32]. For instance, work in [33] conjectures that a scoring function based on physical properties is effective in identifying the native conformation.

Cluster-based decoy selection methods gained dominance over physics-based functions, as the latter were shown not to be consistent in identifying native conformations. The basis of cluster-based methods [10,11,34,35,36,37] is the principle of consensus (that is, conformational similarity) among decoys, as related in Section 1. In addition to varied accuracy, cluster-based methods suffer from time complexity issues, as time increases with the size of the decoy set. Work in [38,39,40] addresses some of these concerns. For instance, a notion of partial clustering is proposed in [41], where not every decoy in the decoy set needs be recognized as a member of a cluster. However, as consensus-based methods, cluster-based decoy selection methods perform poorly when most of the decoys in the decoy set differ much from the known native conformation(s).

Recent complementary lines of research utilize ML models trained on expert-constructed structural features [19,42,43] or discriminate by statistical scoring functions [18,44]. For instance, work in [19] utilizes a state-of-the-art statistical scoring function, GOAP, originally proposed in [44], as an additional feature to convert decoy data into vector data over which a Support Vector Machine (SVM) model is then trained to discriminate between native and non-native conformations. Work in [19] also evaluates ensemble learning over SVM learning, with or without the GOAP-based feature. Four different metrics of evaluation are utilized for the comparison, but no unanimous agreement is reached on the superiority of any method over all metrics. The mixed performance highlights that the utility of ML over statistical scoring functions is yet to be demonstrated.

In [45], a back-propagating neural network-based scoring function was able to provide superior prediction performance over statistical scoring functions. This work suggests the utility of deep learning methods, and more generally of ML methods for decoy selection, but work in this direction needs further evaluation. In principle, ML-based decoy selection promises to advance the state of the art. However, proper application of ML methods also demands addressing overfitting, scarcity of labeled data, imbalanced distribution of data, feature engineering, spurious correlations, and computational complexity as a function of data size.

## 2. Results

We evaluate baseline and novel decoy selection strategies here on 18 proteins of different folds and lengths (number of amino acids), listed in Table 1. They represent easy, medium, and difficult cases for Rosetta. While Rosetta developers argue for a decoy ensemble to be between 10 and 20 K, we generate around 50,000 decoys per target protein on our Mason Argo cluster, so that the size of the decoy set does not influence decoy selection accuracy. The actual size of the decoy set Ω generated for each target is shown in Column 6.

Table 1 shows that Rosetta achieves varied performance on these 18 test cases, as indicated by the minimum distance, min_dist, between generated decoys and a known native conformation in the corresponding PDB entries listed in Column 7; the distance between two conformations is measured via the least Root-Mean-Squared-Deviation (lRMSD), defined in Section 4. The exact delineation of the boundaries between the three categories (easy, medium, hard) of test cases is informed by the performance of cluster-based decoy selection, which we present later as part of a detailed comparative evaluation of different decoy selection strategies.

### 2.1. Evaluation Setup

For a given target protein (listed in Table 1), all decoys with lRMSD from the native conformation (in the corresponding PDB entry in Column 3 in Table 1) within a threshold dist_thresh are deemed native conformations; this threshold allows to populate a positive data set, which is then used to evaluate a decoy selection strategy in terms of the true and false positives it predicts in comparison to the positive data set of native conformations. The threshold dist_thresh is set on a per-target basis, as there are targets on which Rosetta does not get close to 3 Å of the conformation in the target’s PDB entry: If the lowest lRMSD (over all decoys) min_dist ≤0.7 (these are the easy cases in Table 1), dist_thresh is set to 2 Å. Otherwise, dist_thresh is set to the minimum value that results in a non-zero number of native conformations populating the largest-size cluster obtained via leader clustering. For medium-difficulty targets (0.7 Å < min_dist <2 Å), dist_thresh varies in the range 2−4.5 Å. We set the minimum dist_thresh to 6 Å if min_dist ≥2 Å (these are the hard cases). This ensures a non-zero number of native conformations to evaluate decoy selection strategies. The interested reader can find the impact of different values of dist_thresh on the top cluster or basin on each of the target proteins in the Supplementary Material.

We evaluate six decoy selection strategies. The baseline strategy is cluster-based and carries out a simple, follow-the-leader clustering strategy detailed in Section 4. The decoys mapped to the same cluster are within ϵÅin lRMSD of the representative decoy (the first decoy mapped to an empty cluster). We initialize ϵ to be 1 Å and increase this value until we get a non-zero size cluster. For ease of presentation, we will refer to this strategy as Cluster-Size. An alternative selection strategy that informs on the difficulty of each target is proposed, based on drawing uniformly at random. For instance, if the three largest clusters (in terms of the number of decoys in them) revealed by leader clustering have sizes $|C_{1}|$, $|C_{2}|$, and $|C_{3}|$, random drawing yields three groups of sizes $|C_{1}|$, $|C_{2}|$, and $|C_{3}|$, respectively, where the drawing of individuals in a group is uniformly at random over the entire decoy set. Random drawing provides information on whether there is underlying structure in the conformation space sampled by a decoy generation/sampling method such as that used in Rosetta. If a selection strategy offers a subset of decoys as prediction, it is important to understand how much more true information is in that subset over a subset of the same size but drawn at random over the entire decoy set. We will refer to this strategy as Cluster-Random. In our evaluation of Cluster-Random, the decoy set is shuffled, and the process of drawing at random is repeated five separate times/runs; the evaluation employs averages of the metrics described below over the 5 independent runs.

The other four selection strategies are novel ones proposed and described in Section 4. In summary, they all rely on detecting basins in the energy landscape that can be associated with a sampled conformational space. The decoys are grouped into distinct basins (of attraction) in the Rosetta-probed all-atom energy landscape of a specific target sequence via the Structural Bioinformatics Library [46]. As described in Section 4, determination of basins requires specifying a nearest-neighbor distance for which we utilize the same ϵ parameter used in cluster-based selection, similarly initialized to 1 Å. Section 4 also suggests that a filtering mechanism can be employed in the extraction of basins per a persistence threshold. We vary the persistence threshold p_thresh∈[1,10] (higher values means smaller-size basins are merged into larger ones). Uniformly on all test cases, low persistence values ∈[1,3] (that is, close to no filtering) result in better basins (according to our evaluation metrics). Therefore, the evaluation presented below is on basins obtained with close to no persistence-based filtering.

Section 4 introduces various characteristics of basins that are investigated as criteria for selecting basins and offering them as predictions over clusters. Two criteria are shown to be the most important, size and (focal) energy (over persistence and persistence-related characteristics, such as stability; see Section 4 for more details). The size of a basin refers to the number of decoys in it. The energy of a basin is the energy of its focal minimum (the lowest/deepest point in it). Therefore, two basin-based selection strategies are presented and evaluated first, ordering basins by size (from largest to smallest) or by size then energy (from lowest to highest energy). We will refer to them as Basin-Size and Basin-Size+Energy, respectively. Since it is generally unclear how different criteria contribute to accuracy in decoy selection, a multi-objective, Pareto-based approach is employed, where size and energy are treated as possibly competing “optimization” objectives. Based on the concept of dominance along these two objectives, described in detail in Section 4, two additional metrics are associated with a basin, Pareto Rank (PR) and Pareto Count (PC); details are available in Section 4. Therefore, two more basin selection strategies are presented and evaluated, where basins are ordered by their PR (from lowest to highest), or by their PR and PC (basins with the same PR are ordered from highest to lowest PC). We will refer to them as Basin-PR and Basin-PR+PC.

Given a specific sorted ordering (of clusters, or at-random groups, or basins), the top three in the ordering are then analyzed and compared across all six selection strategies on each of the 18 target proteins. The evaluation considers the blind prediction scenario where $G_{1-x}$ groups of decoys are offered as prediction. For instance, $C_{1-x}$ indicates that the top (largest-size) *x* clusters are selected and evaluated. Similarly, $B_{1-x}$ indicates that the top *x* basins (under each of the four possible orderings) are selected and evaluated. The evaluation is limited to x∈[1,3].

#### 2.1.1. Evaluation Metrics

The evaluation presented below focuses on two metrics. The first tracks the percentage of true positives *n*; that is, the percentage of native conformations in $G_{1-x}$ (over the total number of native conformations in the decoy set, per a specific dist_thresh). Focusing on true positives reveals an incomplete view of the performance. The number of false positives is just as, if not more, important. Therefore, the purity metric, *p*, is proposed in this paper, which we define as the proportion of native conformations relative to the size of a group (the purity of $G_{1-x}$ is determined by merging all decoys in $\left\{G_{1},G_{2},\ldots ,G_{x}\right\}$ when x≥1). The inspiration behind this metric is the need to penalize large groups that due to their size may contain a large number of true positives but also contain a high number of false positives. In a setting where the decoys in $G_{1-x}$ are offered as prediction, a reasonable strategy is to draw a smaller, more manageable subset at random from the offered $G_{1-x}$. In the presence of many false positives, the likelihood is low that a native conformation will be obtained via drawing at random. Hence, the need to evaluate the six different selection strategies by the purity of the selected clusters or basins offered as prediction. We note that running time is also an important criterion when evaluating a selection strategy. The comparison of running times is presented in the Supplementary Material, and the results show that the basin-based selection strategies are faster than Cluster-Size.

#### 2.1.2. Experimental Setup

Three sets of results are shown next. First, visualization is employed to compare the six different selection strategies on selected representative targets. A detailed quantitative comparison along the *n* and *p* metrics is presented next. Finally, visualization is employed to expose various characteristics of selected basins by the Pareto-based strategies and to reveal insight into targets where these strategies succeed or fail.

### 2.2. Visual Comparison of Decoy Selection Strategies

**Figure 1:**
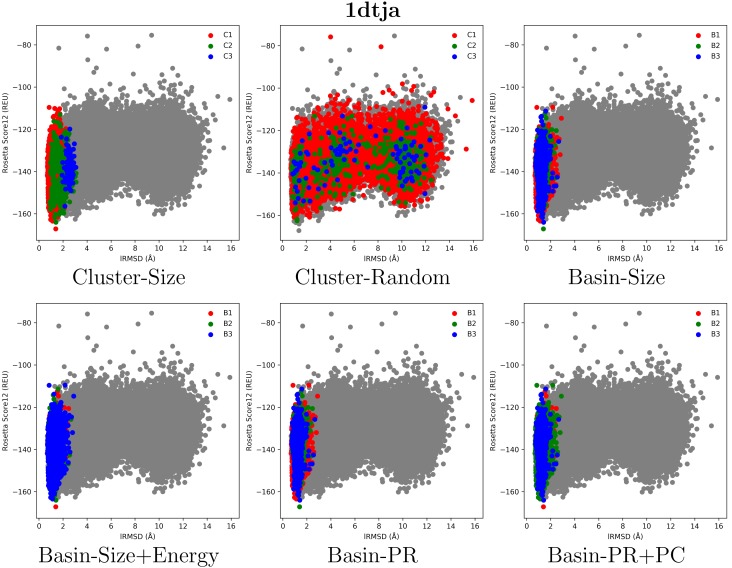
Visualization of selected decoys for the target with PDB entry 1dtja. Decoys are plotted by their lRMSD from the conformation in the PDB entry and their Rosetta score 12 all-atom energy.

**Figure 2:**
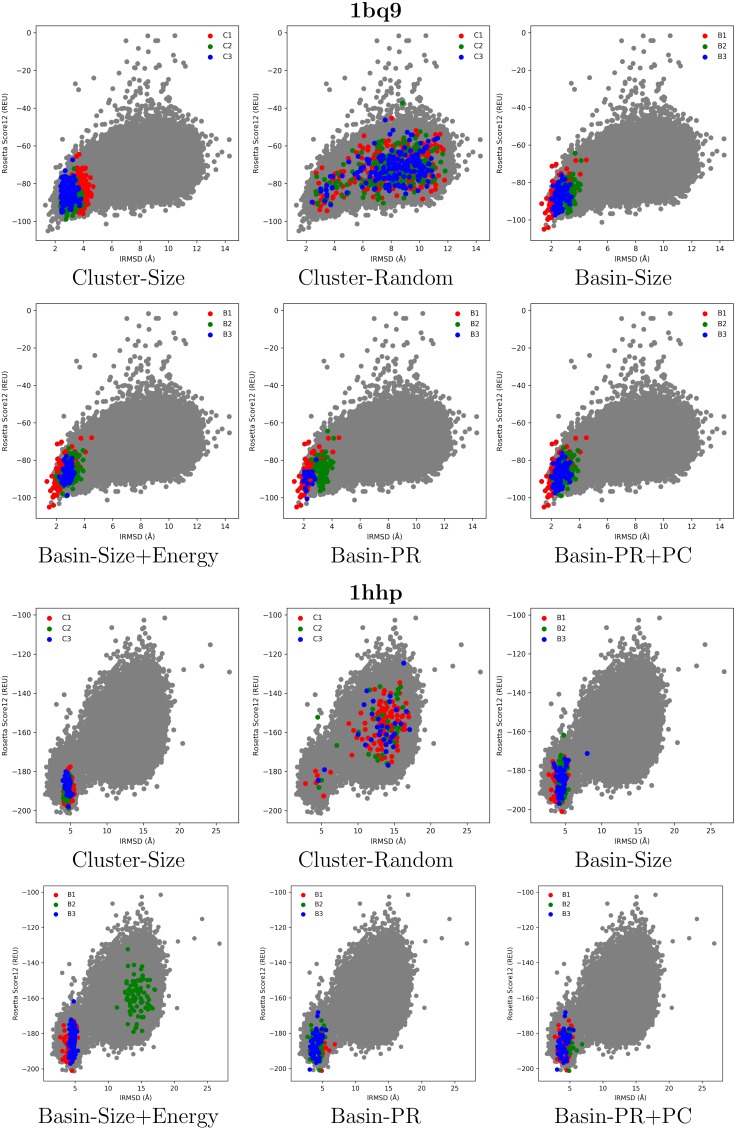
Visualization of selected decoys for targets with PDB entries 1bq9 and 1hhp. Decoys are plotted by their lRMSD from the conformation in the PDB entry and Rosetta score 12 all-atom energy.

**Figure 3:**
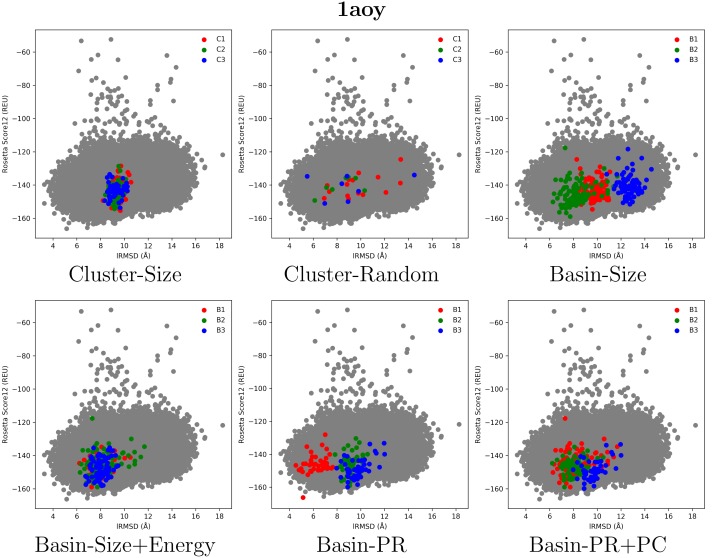
Visualization of selected decoys for the target with PDB entry 1aoy. Decoys are plotted by their lRMSD from the conformation in the PDB entry and Rosetta score 12 all-atom energy.

Figure 1, Figure 2 and Figure 3 select four test cases (1 easy, 2 medium and 1 hard) and show the decoys in each of the top three clusters or basins selected by the various selection strategies. Color coding is used to distinguish the different groups, and decoys are plotted as dots, with the *x* axis tracking the lRMSD of each decoy from the native conformation in the PDB entry of each selected target, and the *y* axis tracking the Rosetta all-atom (score12) energy (measured in Rosetta Energy Units (REUs)).

Figure 1 presents results on an easy case, the protein with PDB entry 1dtja. This target is indeed easy for decoy selection, as the top three groups of decoys selected by all strategies (except Cluster-Random) provide high purity (*p* ranges from 97.2% to 99.6%) with corresponding proportion of near-native conformations (*n* ranges from 19.9% to 97.8%). The top three basins under each basin-based strategy capture similar regions of the decoy space, slightly outperforming Cluster-Size in purity. Cluster-Random performs poorly (*n* ranges from 21.4% to 22.4%, and *p* does not exceed 22.3%), emphasizing the need for an effective selection strategy.

Figure 2 shows results on two medium targets, proteins with PDB entries 1bq9 and 1hhp. In these cases, the top three clusters and basins have many decoys with large lRMSDs from the conformation in the PDB entry (low purity *p* ranging from 1.5% to 80.4%). For instance, in the target with PDB entry 1bq9, the top clusters selected by Cluster-Size suffer from low purity (no decoy with lRMSD <2 Å in any cluster, and purity *p* ranges from 1.5% to 24%). Purity improves in the basin-based selection strategies, ranging from 49.2% to 80.4%. Similar observations hold in the protein with PDB entry 1hhp, where, additionally, the Pareto-based selection strategies improve purity. Figure 2 shows that Basin-Size maps many decoys with high lRMSDs in the second-top basin (color-coded in green). Basin-Size+Energy and the Pareto-based selection strategies improve purity over Basin-Size, which suggests the usefulness of considering energy in decoy selection.

Figure 3 presents results on a hard target, the protein with PDB entry 1aoy. Although all the selection strategies capture mostly decoys with high lRMSDs, thus lowering the purity to 0% in some cases, the Pareto-based strategies fare better. In particular, Basin-PR obtains purity ranging from 21.8% to 78.1%, as the top basin (red) in this selection strategy detects decoys with lRMSD <6 Å. We note that in this hard target, Cluster-Size and Cluster-Random are outperformed by the basin-based selection strategies that take into account the energy landscape. In particular, unlike the basin-based selection strategies, Cluster-Size fails to detect any decoy with lRMSD ≤8 Å.

The highlighted cases suggest that taking into account the energy landscape improves purity, and that Pareto-based strategies may be particularly important on the medium-to-hard cases. We now quantify these observations in the detailed comparison below.

### 2.3. Quantitative Comparison of Decoy Selection Strategies

Table 2, Table 3 and Table 4 compare the six selection strategies on the easy, medium and hard test cases, respectively. As above, the evaluation focuses on *n* and *p* in $G_{1-x}$, where *x* is varied from 1 to 3. The relative size of each $G_{1-x}$ (proportional to the |Ω| decoy set, is also shown for reference).

Table 2 relates the comparative evaluation on the easy test cases. It shows that Cluster-Random performs significantly worse than all other selection strategies (*n* ranges from 4% to 22.4%, and *p* ranges from 6.2% to 22.3%). This indicates, incidentally, that there is structure in the decoy set that can be captured with other, more intelligent decoy selection strategies. In the other four selection strategies, *n* ranges from 1.2% to 97.8%, and *p* ranges from 2.8% to 100%. When focusing on purity *p* alone, the performance of the four proposed basin-based selection strategies is comparable to that of Cluster-Size, with *p* ranging in 48.2–99.9% versus in 2.8–100%. This comparable performance demonstrates that on easy targets clustering is an effective decoy selection strategy.

The comparative evaluation on the medium cases is presented in Table 3. Compared to the easy cases shown in Table 2, there is no medium target where any selection strategy combines both high *p* and high *n*. The best combination is reached on the target with PDB entry 1hz6a (highest n=57.7% combined with corresponding p=58.4%); for reference, on the easy cases, on the protein with PDB entry 1wapa, there is a selection strategy that yields both high *n* and *p* (n=97.6% with p=99.5%). Moreover, on the medium cases, Cluster-Random does slightly better in maintaining a minimum *n* and *p* (n≥0.2% and p≥1.9%) than the other schemes (*n*: 0% and *p*: 0%). However, the upper bound on both metrics is much higher for the other, non-random strategies (n≤57.7% and p≤100%) than Cluster-Random (where n≤18.3% and p≤22.9%). In particular, Basin-PR+PC outperforms all selection strategies in both minimum and maximum *n* while maintaining good purity; minimum n=0.43% with purity p=100% versus minimum n=0% with p=0% in all other non-random strategies, and maximum n=55.5% with p=85.5% versus n=57.7% with p=58.4% in all other non-random strategies.

It is worth noting that the improvement in performance of Cluster-Random (from easy to medium cases) points to little structure in the decoy sets generated by Rosetta for the medium cases. This behavior is at its extreme on the hard cases, shown in Table 4. In these cases, Table 4 shows that Cluster-Random outperforms Cluster-Size by finding more cases of non-zero size clusters with better *n* and *p* (see targets with PDB entries 2ezk, 1aoy, 1isua, and 1aly). Cluster-Size outperforms Cluster-Random on only one case, the protein with PDB entry 1aly. This indicates that there is little structure in the decoy set on the hard cases that a cluster-based selection strategy can leverage, thus exposing in quantitative ways shortcomings in the Rosetta decoy sampling method on these cases and demonstrating the futility of using cluster-based selection strategies on such decoy sets. However, on such challenging decoy sets, the basin-based selection strategies perform better, achieving a maximum *n* of 10% versus Cluster-Size’s *n* of 0.4% and a maximum *p* of 78.1% versus Cluster-Size’s *p* of 42.9%. We note the challenges posed by the hard cases, manifest in the highest n=10% with p=17.8%, which is significantly worse than the best combination achieved on the easy and medium cases.

The quantitative evaluation in Table 2, Table 3 and Table 4 allows drawing a few conclusions. First, Cluster-Random is largely outperformed by Cluster-Size in easy and medium cases but is comparable to Cluster-Size on the hard cases. Cluster-Size is an effective strategy on easy targets (achieving high purity in the targets with PDB entries 1dtdb, 1wapa, 1tig, and 2ci2). On these 4 cases, all basin-based selection strategies do similarly well. On 10/18 cases, Cluster-Size is outperformed by all proposed basin-based selection strategies in terms of purity.

Considering energy does not result in lower purity. On the contrary, in 13/18 cases, selecting by both basin size and energy results in higher or same purity over selecting only by size ($B_{1-3}$ in 1ail, 1dtdb, 1isua, $B_{1}$ in 1dtja, $B_{1}$, $B_{1-2}$ and $B_{1-3}$ in 1c8ca, 2ci2, 1sap, 2ezk, 1aoy $B_{1-2}$ and $B_{1-3}$ in 1bq9, 1hz6a, 1hhp, 1cc5). The effectiveness of the Pareto-based selection strategies can also be observed, particularly as the difficulty of the targets increases. Pareto-based strategies perform better or similarly to Basin-Size and Basin-Size+Energy in 12/18 cases (similarly well in 1dtdb, 1wapa, 1tig, 1dtja, 1hz6a, 1hhp; Basin-PR does better in 1ail, 1aoy, and 1aly; Basin-PR+PC does better in 1c8ca, 1bq9,and 1fwp). Moreover, utilizing PC in addition to PR in Basin-PR+PC improves purity in 7 cases (see $B_{1}$ in 1dtja, 1c8ca, 1hhp, $B_{1-2}$ in 1cc5, $B_{1}$ and $B_{1-2}$ in 2ezk, and $B_{1-2}$ and $B_{1-3}$ in 2ci2, 1bq9).

The steady performance of the Pareto-based selection strategies prompts us now to investigate in further detail the top basins selected by Basin-PR and Basin-PR+PC. We do so on three representative targets (easy, medium, and hard) that showcase hits and misses of the Basin-PR and Basin-PR+PC decoy selection strategies.

### 2.4. Visual Analysis of Pareto-Based Selection Strategies

**Figure 4:**
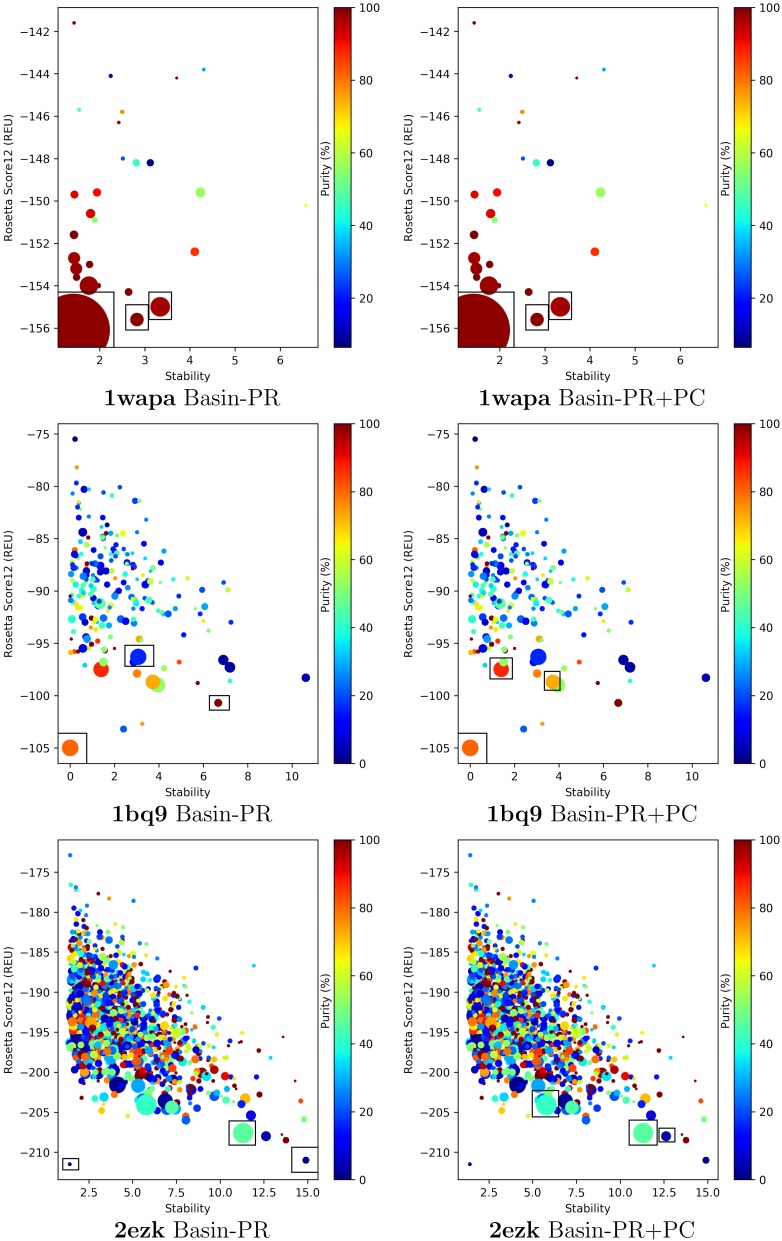
Visualization of basins extracted from the energy landscapes probed for an easy (PDB entry 1wapa), medium (1bq9), and hard target (2ezk). The color-coding scheme varies from blue (low purity) to red (high purity). The size of each disk respects the size of the corresponding basin. Top three basins selected by Basin-PR (left panel) and Basin-PR+PC (right panel) are indicated by encapsulating corresponding disks in rectangles.

We look deeper into the basins selected by Basin-PR and Basin-PR+PC on easy, medium, and hard cases with PDB entries 1wapa, 1bq9, and 2ezk. Several characteristics are depicted for each distinct basin to which decoys are mapped. Figure 4 plots basins as color-coded disks of different sizes. The color shows purity via a color-coding scheme that varies from blue (low purity) to red (high purity). The size of a disk reflects size of the basin it represents. Rectangles encapsulate disks that correspond to basins selected among the top three by the Pareto-based selection strategies.

The *y* axis in Figure 4 tracks the focal energy of each basin, and the *x* axis tracks a complementary characteristic for each basin, stability. Stability is introduced as an additional property of basins in [46] related to persistence. In summary (Section 4 defines stability and its relationship to persistence), a low stability indicates a low-depth basin (relative to the location of the nearest saddle point). Figure 4 reveals that on the easy and medium cases (targets with PDB entries 1wapa and 1bq9), the larger and purer basins tend to appear in the low-energy and low-stability regions. Figure 4 also shows that utilizing PC in addition to PR helps in better selection of basins for the medium and hard cases (ranging from 4% to 46% improvement in purity).

Work in [46] suggests that low-stability basins could be noise and can be filtered out and merged with nearby deeper basins, effectively smoothing the landscape. Figure 4, however, demonstrates that such an approach would worsen performance in the context of decoy selection; In particular, high-purity basins selected by Basin-PR and Basin-PR+PC cover a range of low and high stabilities. This disputes filtering basins by low stability. This is further supported by our evaluation of additional Pareto-based strategies that consider stability as a third objective (in addition to basin size and energy). No improvement is observed; indeed, high-purity basins can be missed when stability is considered (data not shown), as also suggested in Figure 4 on the target with PDB entry 1wapa. Our quantitative evaluation over basins obtained by merging (a process carried out when high persistence values >3 are specified) also shows that purity suffers in return (data not shown). These results are informative. They demonstrate that protein energy landscapes are rugged, and Rosetta all-atom landscapes, as indicated here by low-stability but large and pure basins, can be exceptionally rugged.

## 3. Discussion

The results presented in this paper suggest that basins in the energy landscape probed by a template-free structure prediction method can be leveraged for decoy selection. In particular, while energy is often ignored in favor of conformational similarity in cluster-based selection strategies, the presented work indicates that energy can be employed reliably to improve decoy selection. The results support that selection of basins is more effective than selection of clusters for decoy selection. Considering not just the size but also the energy of a basin in selection is more effective in yielding high-purity basins containing a low number of false positives. In particular, Pareto-based selection strategies demonstrate better performance on a variety of targets that include hard cases with conformation spaces poorly sampled by the Rosetta decoy sampling method.

On easy targets, comparable performance is obtained by cluster- and basin-based selection strategies. On such targets, drawing at random performs worst, indicating the presence of structure in the Rosetta-generated decoy set that is then leveraged by cluster- or basin-based selection strategies. Such structure is progressively weakened on the medium and hard cases, where drawing at random approaches and even surpasses the performance of cluster-based selection. However, even in such cases, the basin-based selection strategies perform well. In many of the hard cases, the Pareto-based strategies achieve the best performance, particularly in terms of purity. This is an important contribution of this paper, as it suggests a landscape-based view of selecting decoys can lower the number of false positives (non-native decoys) reported.

As shown in the Supplementary Material, the running times of the basin-based selection strategies (including the time to compute basins) do not exceed and are actually lower than the time it takes to cluster conformations by structural similarity (via leader clustering). In total, the running time of computing and selecting basins via any of the four basin-based strategies proposed in this paper is a few hours on one CPU. One can decrease the running time by some a-priori filtering of conformations. A reasonable strategy is to do so based on energy. As we describe in detail in the Supplementary Material, one has to be careful when doing so. Removing an arbitrary percentage of the lowest-energy conformations may seem appealing and justified as a landscape-smoothing strategy. However, as the results in the Supplementary Material indicate, such a strategy removes focal energies, changes the structure of the landscape, and results in many spurious basins. On the other hand, as also shown in the Supplementary Material, removing high-energy conformations instead retains more of the structure of the landscape to allow the basin-based selection strategies to detect and select true basins in the landscape.

The presented work opens many lines of enquiry to address current limitations. For instance, while cluster- and basin-based selection strategies may be useful for ranking, they do not provide a reliable estimate of the quality of a single decoy. On the other hand, by considering the energy landscape as a whole, the decoys in top basins provide an informative set that can be inspected by statistical scoring functions to reveal indicators of nativeness in the presence of the multiplicity of native conformations. Moreover, while the evaluation presented in this paper focuses on the Rosetta all-atom energy landscape, in principle, all the concepts and techniques proposed in this paper extend to landscapes of any scoring function, including statistical functions. These lines of enquiry, while beyond the scope of the work presented here, promise to advance the state of decoy selection.

## 4. Materials and Methods

### 4.1. Energy-Less Decoy Selection

As related in Section 1 and Section 2, the predominant decoy selection approach in CASP for over a decade has been cluster-based selection. For the purpose of a baseline, cluster-based selection strategy to which we can compare the proposed basin-based selection strategies, we implement leader clustering. Leader clustering implements a follow-the-leader approach. It is an order-dependent, incremental clustering algorithm useful for clustering large datasets. The decoys are shuffled first. In the resulting order, each decoy either form a new cluster (becoming its representative) or is assigned to the first cluster whose representative (the first decoy mapped to it) is within ϵÅ in lRMSD. Briefly, lRMSD removes differences due to rigid-body motions, and reports the RMSD (a weighted variant of Euclidean distance) after an optimal superimposition that minimizes RMSD [47]. To reduce computational costs, the decoys are first superimposed onto an arbitrarily-chosen reference one (we select the first decoy in the decoy set to serve as reference), and then only RMSD is used to determine the distance between any two decoys.

As Section 2 shows, an additional selection strategy employed in our evaluation is Cluster-Random, which does not cluster decoys in any meaningful way, but draws decoys uniformly at random from the decoy set, bounded by the sizes of the *x* largest clusters revealed by leader clustering (*x* is a parameter considered in the evaluation presented in Section 2).

### 4.2. Energy (Landscape)-Based Decoy Selection

The other four, novel selection strategies leverage the concept of basins in the energy landscape of a protein. Before relating these strategies, we first define the terms of an energy landscape and basins. We then proceed to describe how basins are identified in the landscape, and how *x* basins are selected among the identified ones for the purpose of addressing decoy selection.

#### 4.2.1. Energy Landscapes

A molecular energy landscape is a specific instance of a fitness landscape, a concept that originated in theoretical biology more than eighty years ago [48] but has since become a useful construction in diverse scientific disciplines, from the physics of disordered systems such as spin-glasses, molecular biology [22], characterization of optimization problems in AI [7], and the broader study of complex systems [49]. Specifically, a fitness landscape consists of a set *X* of points, a notion N(X) of neighborhood, distance, or accessibility on *X*, and a fitness function $f:X\to R_{\geq 0}$ that assigns a fitness to every x∈X. The neighborhood function N:X→P(X) assigns neighbors N(x) to every x∈X. In our context, points x∈X are decoy conformations, and the fitness function scores decoys.

A fitness landscape can be high-dimensional and multimodal. It may contain many local structures, such as basins and basin-separating barriers. In molecular (energy) landscapes, a basin corresponds to a long-lived, thermodynamically-stable or semi-stable state [22]. The notion of a basin is tied to a local, focal optimum: a local optimum in the landscape is surrounded by a basin of attraction, which is the set of points on the landscape from which steepest descent/ascent converges to that focal optimum. In molecular landscapes, the focus is on local minima. In lieu of observing a molecule rearranging itself between conformations and reaching a local minimum, one can enrich the landscape with connectivity information to identify focal minima and their basins, as proposed in recent work [46].

#### 4.2.2. Elucidating Basins via Graph Embeddings of Landscapes

Consider a decoy set Ω generated by a decoy sampling method. Ω can be embedded in a nearest-neighbor graph (nngraph) G=(V,E) as follows. The vertex set *V* is populated with the decoys; that is, V←Ω. The edge set *E* is populated by inferring the neighborhood structure of the landscape. Given a selected distance function measuring the distance between two decoys (e.g., lRMSD), each vertex u∈V is connected to other vertices v∈V if d(u,v)≤ϵ, with ϵ being a user-defined parameter. A small ϵ may result in a disconnected graph in the presence of non-uniform sampling of the landscape. This can be remedied by increasing ϵ or the number of nearest neighbors of *u*.

The resulting nngraph can be analyzed to detect local minima. Let u∈V, and let v∈N(u), where N(u) denotes the neighborhood of *u*. *u* is a local minimum if ∀v∈Vf(u)≤f(v). Each local minimum becomes a focal minimum of some basin. The rest of the vertices are assigned to basins as follows. Each vertex *u* is associated a negative gradient estimated by selecting the edge (u,v) that maximizes the ratio [f(u)−f(v)]/d(u,v). From each vertex *u* that is not a local minimum, the negative gradient is iteratively followed (i.e., the edge that maximizes the above ratio is selected and followed) until a local minimum is reached. Vertices that via this process reach the same local minimum are assigned to the basin associated with that minimum.

#### 4.2.3. Characteristics of Basins

Several characteristics can be associated with each identified basin, such as size, focal energy, persistence, and stability. Basin size refers to the number of decoys assigned to the same basin. The energy of a basin is the energy of its focal minimum (its deepest point). Basin persistence is a concept used in spatial statistics to filter out basins possibly attributed to noisy fitness/energy functions [46]. The persistence of a basin *B* is f(saddle)−f(B), where f(B) refers to the focal energy of *B*. A (pseudo-)saddle is identified as a vertex *u* from which the iterative process of following the negative gradient, described above, leads to the focal minimum of *B* but has a neighbor *v* with f(v)<f(u) from which the iterative process leads a different local minimum. Persistence measures how shallow a basin is. In spatial statistics, a persistence threshold p_thresh can be specified as a way of retaining only basins with persistence above p_thresh (merging those with lower persistence into the surviving basins). Persistence gives rise to another related concept, stability. Stability can be measured by embedding the persistence of each basin in a two-dimensional graph with the focal energy of a basin on the *x* axis and the fitness/energy of the corresponding saddle on the *y* axis. The distance of each point (corresponding to a basin in this plot), from the identity line measures the stability of the corresponding basin.

#### 4.2.4. Basin-Based Selection Strategies

The characteristics described above can be leveraged to select basins and offer them as prediction in the context of decoy selection. For instance, one can order basins by their size, from largest to smallest. We refer to this strategy as Basin-Size. In contrast, one can also consider the focal energy of a basin in the selection process. For instance, after sorting basins by size, the top ten basins in the sorted order can be reordered by their energy (from low to high focal energy). The top *x* basins in this sorted order are then selected. We refer to this strategy as Basin-Size+Energy.

The two other decoy selection schemes, Basin-Size and Basin-Size+Energy, uses the two properties of basins (size, depth/energy) in detecting near-native structures. We also utilize these properties from a different perspective in two other selection schemes: Pareto rank and Pareto rank + count. These two techniques stem from the concept of Pareto optimality applied to multi-objective optimization problems. As noted in Section 2, the other two characteristics, persistence and stability, which are related to each-other, do not improve decoy selection and do not provide any clear sorted order that can be utilized to select basins.

### 4.3. Multi-Objective, Pareto-Based Basin Selection Strategies

In addition to selecting basins by size or by size and energy, we propose two more basin selection strategies that acknowledge the unclear interactions between basin size and energy. Specifically, size and negative of energy are considered as two separate optimization objectives, and the problem of basin selection is treated as a multi-objective optimization problem. Specifically, a solution to a multi-objective problem seeks to optimize two or more conflicting objectives. In this scenario, Pareto-optimal solutions are sought, as a single solution minimizing all conflicting objectives simultaneously is typically non-existent. A Pareto-optimal solution cannot be improved in one objective without sacrificing the quality of at least one other objective. In other words, a solution $S_{1}$ Pareto-dominates another solution $S_{2}$ if the following two conditions are satisfied.

1. For all optimization objectives *i*, $score_{i}\left(S_{1}\right)\geq score_{i}\left(S_{2}\right)$
2. For at least one optimization objective *i*, $score_{i}\left(S_{1}\right)>score_{i}\left(S_{2}\right)$

We consider strong dominance (replacing ≥ with > in the above definition) on basin size and (negative) energy. One can now associate two additional quantities with each basin, Pareto Rank (PR) and Pareto Count (PC). The PR of a basin *B* is the number of basins that dominate *B*. The PC of a basin *B* is the number of basins that *B* dominates. Two additional, Pareto-based selection strategies are now proposed. In Basin-PR, the basins are sorted by low to high PR values, and the top *x* basins in this sorted order are selected and analyzed, as presented in Section 2. In Basin-PR+PC, PC is additionally considered. Basins with the same PR value are sorted from high to low PCs, and the top *x* basins in this resulting sorted order are selected and analyzed, as also related in Section 2.

## Figures and Tables

**Table 1:** Testing dataset (* denotes proteins with a predominant β fold and a short helix).

		PDB ID	Fold	Length	Ω	min_dist (Å)
Easy	1.	1ail	α	70	53,568	0.50
	2.	1dtdb	α+β	61	57,839	0.51
	3.	1wapa	β	68	51,841	0.60
	4.	1tig	α+β	88	52,099	0.60
	5.	1dtja	α+β	74	53,526	0.68
Medium	6.	1hz6a	α+β	64	57,474	0.72
	7.	1c8ca	β *	64	53,322	1.08
	8.	2ci2	α+β	65	52,220	1.21
	9.	1bq9	β	53	53,663	1.30
	10.	1hhp	β *	99	52,159	1.52
	11.	1fwp	α+β	69	53,133	1.56
	12.	1sap	β	66	51,209	1.75
Hard	13.	2h5nd	α	123	51,475	2.00
	14.	2ezk	α	93	50,192	2.56
	15.	1aoy	α	78	52,218	3.26
	16.	1cc5	α	83	51,687	3.95
	17.	1isua	coil	62	60,360	5.53
	18.	1aly	β	146	53,274	8.53

**Table 2:** Comparison of all selection strategies on the easy cases.

		1ail	1dtdb	1wapa	1tig	1dtja
Cluster-Random	C${}_{1}$	n: 4%	n: 17.8%	n: 5.2%	n: 8.8%	n: 21.4%
		p: 6.2%	p: 18.2%	p: 10.1%	p: 15.2%	p: 22.3%
		s: 4.1%	s: 22.3%	s: 5.2%	s: 8.7%	s: 21.6%
	C${}_{1-2}$	n: 6.6%	n: 18.6%	n: 9.8%	n: 11.3%	n: 22.2%
		p: 6.3%	p: 18.2%	p: 10%	p: 15.2%	p: 22.2%
		s: 6.7%	s: 23.3%	s: 10%	s: 11.2%	s: 22.4%
	C${}_{1-3}$	n: 8.5%	n: 19.1%	n: 10%	n: 13.6%	n: 22.4%
		p: 6.3%	p: 18.2%	p: 10%	p: 15.1%	p: 22.3%
		s: 8.7%	s: 23.9%	s: 10.2%	s: 13.6%	s: 22.6%
Cluster-Size	C${}_{1}$	n: 63.9%	n: 97.6%	n: 50.8%	n: 57.3%	n: 95.5%
		p: 99.5%	p: 99.9%	p: 99.9%	p: 99.1%	p: 99.2%
		s: 4.1%	s: 22.3%	s: 5.2%	s: 8.7%	s: 21.6%
	C${}_{1-2}$	n: 64.4%	n: 97.6%	n: 97.6%	n: 73%	n: 97.8%
		p: 61.1%	p: 95.7%	p: 99.5%	p: 98.2%	p: 98%
		s: 6.7%	s: 23.3%	s: 10%	s: 11.2%	s: 22.4%
	C${}_{1-3}$	n: 65.6%	n: 97.6%	n: 97.6%	n: 88.4%	n: 97.8%
		p: 48.2%	p: 93.3%	p: 97.3%	p: 98.4%	p: 97.2%
		s: 8.7%	s: 23.9%	s: 10.2%	s: 13.6%	s: 22.6%
Basin-Size	B${}_{1}$	n: 47.2%	n: 85.3%	n: 76.8%	n: 28.8%	n: 36.9%
		p: 100%	p: 99%	p: 98.9%	p: 100%	p: 98.9%
		s: 3%	s: 19.7%	s: 7.9%	s: 4.4%	s: 8.4%
	B${}_{1-2}$	n: 48.4%	n: 94.9%	n: 81.8%	n: 40.1%	n: 56.7%
		p: 52.8%	p: 98.9%	p: 98.8%	p: 99.6%	p: 99.1%
		s: 5.8%	s: 21.9%	s: 8.4%	s: 6.1%	s: 12.8%
	B${}_{1-3}$	n: 48.4%	n: 94.9%	n: 86.3%	n: 50.2%	n: 70.7%
		p: 44.8%	p: 94.8%	p: 98.7%	p: 99.7%	p: 99.2%
		s: 6.9%	s: 22.9%	s: 8.9%	s: 7.6%	s: 16%
Basin-Size+Energy	B${}_{1}$	n: 1.2%	n: 85.3%	n: 76.8%	n: 2.7%	n: 19.9%
		p: 2.8%	p: 99%	p: 98.9%	p: 88.4%	p: 99.6%
		s: 3%	s: 19.7%	s: 7.9%	s: 0.5%	s: 4.5%
	B${}_{1-2}$	n: 48.4%	n: 94.9%	n: 79.1%	n: 31.5%	n: 33.8%
		p: 52.8%	p: 98.9%	p: 98.9%	p: 98.9%	p: 99.6%
		s: 5.8%	s: 21.9%	s: 8.2%	s: 4.8%	s: 7.6%
	B${}_{1-3}$	n: 61.9%	n: 95.9%	n: 84.1%	n: 42.8%	n: 70.7%
		p: 58.6%	p: 98.9%	p: 98.8%	p: 98.8%	p: 99.2%
		s: 6.7%	s: 22.1%	s: 8.7%	s: 6.5%	s: 16%
Basin-PR	B${}_{1}$	n: 47.2%	n: 85.3%	n: 76.8%	n: 28.8%	n: 36.9%
		p: 100%	p: 99%	p: 98.9%	p: 100%	p: 98.9%
		s: 3%	s: 19.7%	s: 7.9%	s: 4.4%	s: 8.4%
	B${}_{1-2}$	n: 48.4%	n: 94.9%	n: 79.1%	n: 31.5%	n: 56.7%
		p: 52.8%	p: 98.9%	p: 98.9%	p: 98.9%	p: 99.1%
		s: 5.8%	s: 21.9%	s: 8.2%	s: 4.8%	s: 12.8%
	B${}_{1-3}$	n: 61.9%	n: 94.9%	n: 84.1%	n: 42.8%	n: 70.7%
		p: 58.6%	p: 98.9%	p: 98.8%	p: 98.8%	p: 99.2%
		s: 6.7%	s: 21.9%	s: 8.7%	s: 6.6%	s: 16%
Basin-PR+PC	B${}_{1}$	n: 47.2%	n: 85.3%	n: 76.8%	n: 28.8%	n: 19.9%
		p: 100%	p: 99%	p: 98.9%	p: 100%	p: 99.6%
		s: 3%	s: 19.7%	s: 7.9%	s: 4.4%	s: 4.5%
	B${}_{1-2}$	n: 48.4%	n: 94.9%	n: 81.8%	n: 31.5%	n: 56.7%
		p: 52.8%	p: 98.9%	p: 98.8%	p: 98.9%	p: 99.1%
		s: 5.8%	s: 21.9%	s: 8.4%	s: 4.8%	s: 12.8%
	B${}_{1-3}$	n: 61.9%	n: 95.4%	n: 84.1%	n: 42.8%	n: 70.7%
		p: 58.6%	p: 98.8%	p: 98.8%	p: 98.8%	p: 99.2%
		s: 6.7%	s: 22%	s: 8.7%	s: 6.6%	s: 16%

**Table 3:** Comparison of all selection strategies on the medium cases.

		1hz6a	1c8ca	2ci2	1bq9	1hhp	1fwp	1sap
Cluster-Random	C${}_{1}$	n: 4.5%	n: 3.5%	n: 0.4%	n: 0.8%	n: 0.2%	n: 1.9%	n: 9.5%
		p: 11.4%	p: 11.4%	p: 22.5%	p: 1.9%	p: 2.8%	p: 6%	p: 2.3%
		s: 4.4%	s: 3.4%	s: 0.4%	s: 0.6%	s: 0.2%	s: 1.8%	s: 9.3%
	C${}_{1-2}$	n: 7.7%	n: 5.3%	n: 0.6%	n: 1.4%	n: 0.3%	n: 3.2%	n: 14.6%
		p: 11.3%	p: 11.2%	p: 22.9%	p: 2.1%	p: 2.7%	p: 6.1%	p: 2.4%
		s: 7.7%	s: 5.2%	s: 0.6%	s: 1%	s: 0.3%	s: 3.1%	s: 13.9%
	C${}_{1-3}$	n: 10.9%	n: 6.3%	n: 0.8%	n: 1.9%	n: 0.3%	n: 4%	n: 18.3%
		p: 11.4%	p: 11.2%	p: 22.2%	p: 2.1%	p: 2.3%	p: 5.8%	p: 7.4%
		s: 10.8%	s: 6.2%	s: 0.8%	s: 1.4%	s: 0.3%	s: 4%	s: 17.4%
Cluster-Size	C${}_{1}$	n: 0%	n: 10%	n: 1.3%	n: 0.6%	n: 1.5%	n: 29.1%	n: 0%
		p: 0%	p: 32.1%	p: 82%	p: 1.5%	p: 19.8%	p: 92.8%	p: 0%
		s: 4.4%	s: 3.4%	s: 0.4%	s: 0.64%	s: 0.19%	s: 1.8%	s: 9.3%
	C${}_{1-2}$	n: 0%	n: 11.8%	n: 2.4%	n: 9.1%	n: 2.6%	n: 36.3%	n: 44.1%
		p: 0%	p: 24.7%	p: 89.4%	p: 13.6%	p: 25.4%	p: 69.2%	p: 7.3%
		s: 7.7%	s: 5.2%	s: 0.6%	s: 1.04%	s: 0.26%	s: 3.1%	s: 13.9%
	C${}_{1-3}$	n: 26.4%	n: 20.5%	n: 3.2%	n: 21%	n: 3.7%	n: 44.1%	n: 55.9
		p: 27.7%	p: 36.3%	p: 92%	p: 24%	p: 28.7%	p: 63.7%	p: 7.4
		s: 10.8%	s: 6.2%	s: 0.8%	s: 1.4%	s: 0.32%	s: 4%	s: 17.4%
Basin-Size	B${}_{1}$	n: 55.5%	n: 6.1%	n: 0.3%	n: 9.3%	n: 3.5%	n: 5.6%	n: 0%
		p: 85.5%	p: 32.9%	p: 47.2%	p: 80.4%	p: 53.6%	p: 97.7%	p: 0%
		s: 7.3%	s: 2%	s: 0.13%	s: 0.18%	s: 0.16%	s: 0.33%	s: 4.4%
	B${}_{1-2}$	n: 55.5%	n: 20.2%	n: 0.3%	n: 11.1%	n: 3.5%	n: 9.1%	n: 32.4%
		p: 50%	p: 60.8%	p: 23.6%	p: 49.2%	p: 27%	p: 97.2%	p: 9.3%
		s: 12.6%	s: 3.6%	s: 0.3%	s: 0.4%	s: 0.32%	s: 0.54%	s: 8.1%
	B${}_{1-3}$	n: 55.5%	n: 22.3%	n: 0.3%	n: 19.8%	n: 5.6%	n: 10.7%	n: 51.4%
		p: 39.3%	p: 48.5%	p: 15.9%	p: 60.8%	p: 30.8%	p: 84.2%	p: 11.5%
		s: 16%	s: 5%	s: 0.4%	s: 0.51%	s: 0.45%	s: 0.74%	s: 10.3%
Basin-Size+Energy	B${}_{1}$	n: 55.5%	n: 3.3%	n: 0.42%	n: 9.3%	n: 3.5%	n: 3.5%	n: 32.4%
		p: 85.5%	p: 47.8%	p: 100%	p: 80.4%	p: 53.6%	p: 96.4%	p: 20.2%
		s: 7.3%	s: 0.8%	s: 0.1%	s: 0.18%	s: 0.16%	s: 0.21%	s: 3.7%
	B${}_{1-2}$	n: 55.5%	n: 17.4%	n: 0.71%	n: 14.1%	n: 5.6%	n: 3.7%	n: 51.4%
		p: 66.6%	p: 80.6%	p: 68.9%	p: 68.2%	p: 47.7%	p: 58.4%	p: 20%
		s: 9.4%	s: 2.4%	s: 0.23%	s: 0.32%	s: 0.29%	s: 0.37%	s: 5.9%
	B${}_{1-3}$	n: 55.7%	n: 20.1%	n: 1.13%	n: 20.5%	n: 8.5%	n: 9.3%	n: 51.4%
		p: 55.7%	p: 80.4%	p: 76.9%	p: 69.6%	p: 51.4%	p: 77%	p: 18.2%
		s: 11.3%	s: 2.7%	s: 0.33%	s: 0.46%	s: 0.41%	s: 0.7%	s: 6.5%
Basin-PR	B${}_{1}$	n: 55.5%	n: 3.3%	n: 0.1%	n: 9.3%	n: 0.1%	n: 3.5%	n: 32.4%
		p: 85.5%	p: 47.8%	p: 100%	p: 80.4%	p: 5%	p: 96.4%	p: 20.2%
		s: 7.3%	s: 0.8%	s: 0.01%	s: 0.18%	s: 0.04%	s: 0.21%	s: 3.7%
	B${}_{1-2}$	n: 55.5%	n: 17.4%	n: 0.1%	n: 11.1%	n: 3.6%	n: 9.1%	n: 32.4%
		p: 58.3%	p: 80.6%	p: 7.7%	p: 49.2%	p: 44.2%	p: 97.2%	p: 9.3%
		s: 10.8%	s: 2.4%	s: 0.15%	s: 0.35%	s: 0.2%	s: 0.54%	s: 8.1%
	B${}_{1-3}$	n: 57.7%	n: 23.5%	n: 0.3%	n: 13.3%	n: 6.9%	n: 9.3%	n: 51.4%
		p: 58.4%	p: 58.5%	p: 26.5%	p: 53.9%	p: 55.6%	p: 77%	p: 11.5%
		s: 11.2%	s: 4.4%	s: 0.2%	s: 0.51%	s: 0.31%	s: 0.7%	s: 10.3%
Basin-PR+PC	B${}_{1}$	n: 55.5%	n: 14%	n: 0.43%	n: 9.3%	n: 3.5%	n: 3.5%	n: 32.4%
		p: 85.5%	p: 96.3%	p: 100%	p: 80.4%	p: 53.6%	p: 96.4%	p: 20.2%
		s: 7.3%	s: 1.6%	s: 0.1%	s: 0.18%	s: 0.16%	s: 0.21%	s: 3.7%
	B${}_{1-2}$	n: 55.5%	n: 17.4%	n: 0.72%	n: 14.1%	n: 3.6%	n: 9.1%	n: 32.4%
		p: 50%	p: 80.6%	p: 68.9%	p: 68.2%	p: 44.2%	p: 97.2%	p: 9.3%
		s: 12.6%	s: 2.4%	s: 0.23%	s: 0.32%	s: 0.2%	s: 0.54%	s: 8.1%
	B${}_{1-3}$	n: 55.5%	n: 23.5%	n: 0.93%	n: 22.7%	n: 6.9%	n: 9.3%	n: 51.4%
		p: 39.3%	p: 58.5%	p: 67.7%	p: 74.3%	p: 55.6%	p: 77%	p: 11.5%
		s: 16%	s: 4.4%	s: 0.31%	s: 0.46%	s: 0.31%	s: 0.7%	s: 10.3%

**Table 4:** Comparison of all selection strategies on the hard cases.

		2h5nd	2ezk	1aoy	1cc5	1isua	1aly
Cluster-Random	C${}_{1}$	n: 0%	n: 0.01%	n: 0.02%	n: 0%	n: 0.02%	n: 0%
		p: 0%	p: 5%	p: 8.0%	p: 0%	p: 5.5%	p: 0%
		s: 0.004%	s: 0.02%	s: 0.03%	s: 0.01%	s: 0.02%	s: 0.01%
	C${}_{1-2}$	n: 0%	n: 0.03%	n: 0.03%	n: 0%	n: 0.04%	n: 0%
		p: 0%	p: 7.5%	p: 8.2%	p: 0%	p: 6%	p: 0%
		s: 0.008%	s: 0.05%	s: 0.04%	s: 0.02%	s: 0.03%	s: 0.02%
	C${}_{1-3}$	n: 0%	n: 0.05%	n: 0.04%	n: 0%	n: 0.04%	n: 0.01%
		p: 0%	p: 10%	p: 6.9%	p: 0%	p: 5%	p: 1.4%
		s: 0.01%	s: 0.07%	s: 0.06%	s: 0.03%	s: 0.05%	s: 0.03%
Cluster-Size	C${}_{1}$	n: 0%	n: 0%	n: 0%	n: 0%	n: 0%	n: 0%
		p: 0%	p: 0%	p: 0%	p: 0%	p: 0%	p: 0%
		s: 0.004%	s: 0.02%	s: 0.03%	s: 0.01%	s: 0.02%	s: 0.01%
	C${}_{1-2}$	n: 0%	n: 0%	n: 0%	n: 0%	n: 0%	n: 0.3%
		p: 0%	p: 0%	p: 0%	p: 0%	p: 0%	p: 40%
		s: 0.008%	s: 0.05%	s: 0.04%	s: 0.02%	s: 0.03%	s: 0.02%
	C${}_{1-3}$	n: 0%	n: 0%	n: 0%	n: 0%	n: 0%	n: 0.4%
		p: 0%	p: 0%	p: 0%	p: 0%	p: 0%	p: 42.9%
		s: 0.01%	s: 0.07%	s: 0.06%	s: 0.03%	s: 0.05%	s: 0.03%
Basin-Size	B${}_{1}$	n: 0%	n: 0.96%	n: 0%	n: 0.03%	n: 0.34%	n: 0%
		p: 0%	p: 41.2%	p: 0%	p: 1.14%	p: 14.1%	p: 0%
		s: 0.27%	s: 0.3%	s: 0.2%	s: 0.17%	s: 0.13%	s: 0.06%
	B${}_{1-2}$	n: 0%	n: 2%	n: 0.2%	n: 0.03%	n: 0.34%	n: 0.07%
		p: 0%	p: 43.5%	p: 4.9%	p: 0.6%	p: 7.1%	p: 1.6%
		s: 0.38%	s: 0.6%	s: 0.39%	s: 0.32%	s: 0.26%	s: 0.12%
	B${}_{1-3}$	n: 10%	n: 2%	n: 0.2%	n: 0.03%	n: 0.34%	n: 0.07%
		p: 17.4%	p: 33%	p: 3.4%	p: 0.42%	p: 4.9%	p: 1.1%
		s: 0.48%	s: 0.8%	s: 0.57%	s: 0.46%	s: 0.38%	s: 0.17%
Basin-Size+Energy	B${}_{1}$	n: 0%	n: 1.02%	n: 0.05%	n: 0%	n: 0.34%	n: 0%
		p: 0%	p: 45.9%	p: 3.5%	p: 0%	p: 14.1%	p: 0%
		s: 0.09%	s: 0.29%	s: 0.16%	s: 0.14%	s: 0.13%	s: 0.05%
	B${}_{1-2}$	n: 0%	n: 1.5%	n: 0.23%	n: 1.15%	n: 0.34%	n: 0%
		p: 0%	p: 45.7%	p: 6.9%	p: 27.3%	p: 7.6%	p: 0%
		s: 0.37%	s: 0.41%	s: 0.36%	s: 0.23%	s: 0.24%	s: 0.1%
	B${}_{1-3}$	n: 10%	n: 2.4%	n: 0.28%	n: 1.2%	n: 0.44%	n: 0%
		p: 17.8%	p: 43.8%	p: 6.1%	p: 18.9%	p: 6.6%	p: 0%
		s: 0.47%	s: 0.72%	s: 0.51%	s: 0.35%	s: 0.35%	s: 0.16%
Basin-PR	B${}_{1}$	n: 0%	n: 0%	n: 0.56%	n: 0.03%	n: 0%	n: 0.27%
		p: 0%	p: 0%	p: 78.1%	p: 1.14%	p: 0%	p: 40%
		s: 0.006%	s: 0.03%	s: 0.08%	s: 0.17%	s: 0.02%	s: 0.02%
	B${}_{1-2}$	n: 0%	n: 1.02%	n: 0.56%	n: 0.03%	n: 0%	n: 0.27%
		p: 0%	p: 41.9%	p: 33%	p: 1.12%	p: 0%	p: 19.1%
		s: 0.28%	s: 0.32%	s: 0.19%	s: 0.17%	s: 0.12%	s: 0.04%
	B${}_{1-3}$	n: 0%	n: 1.02%	n: 0.56%	n: 0.66%	n: 0.07%	n: 0.27%
		p: 0%	p: 41.1%	p: 21.8%	p: 15.8%	p: 4.8%	p: 8%
		s: 0.31%	s: 0.32%	s: 0.28%	s: 0.23%	s: 0.21%	s: 0.09%
Basin-PR+PC	B${}_{1}$	n: 0%	n: 1.02%	n: 0.18%	n: 0%	n: 0%	n: 0%
		p: 0%	p: 45.9%	p: 9.8%	p: 0%	p: 0%	p: 0%
		s: 0.27%	s: 0.29%	s: 0.2%	s: 0.14%	s: 0.05%	s: 0.04%
	B${}_{1-2}$	n: 0%	n: 2%	n: 0.23%	n: 0.63%	n: 0%	n: 0%
		p: 0%	p: 43.5%	p: 6.9%	p: 17.5%	p: 0%	p: 0%
		s: 0.37%	s: 0.6%	s: 0.36%	s: 0.2%	s: 0.11%	s: 0.08%
	B${}_{1-3}$	n: 0%	n: 2.0%	n: 0.23%	n: 0.73%	n: 0.03%	n: 0%
		p: 0%	p: 39.7%	p: 5.5%	p: 15.8%	p: 1.2%	p: 0%
		s: 0.39%	s: 0.66%	s: 0.46%	s: 0.26%	s: 0.14%	s: 0.10%

## Supplementary Materials

The following are available online, Figure S1: title, Table S1: title, Video S1: title.

## Abbreviations

The following abbreviations are used in this manuscript:

PDB	Protein Data Bank
CASP	Critical Assessment of protein Structure Prediction
lRMSD	least Root-Mean-Squared-Deviation
ML	Machine Learning
PC	Pareto Count
PR	Pareto Rank
SVM	Support Vector Machines
