# Supplementary material for: From Extraction of Local Structures of Protein Energy Landscapes to Improved Decoy Selection in Template-Free Protein Structure Prediction
Source: Molecules. 2018 Jan 19;23(1):216. doi: 10.3390/molecules23010216 (PMC6017496; doi:10.3390/molecules23010216)
Supplement: Supplementary file 1 [file molecules-23-00216-s001.pdf]

Supplementary Material

From Extraction of Local Structures of Protein Energy Landscapes to Improved Decoy Selection in Template-free Protein Structure Prediction

Nasrin Akhter[1] and Amarda Shehu[1,2,3,*]
[1]Department of Computer Science, [2]Department of Bioengineering, [3]School of Systems Biology
George Mason University , Fairfax, VA, USA
*E-mail: Corresponding author [amarda]@gmu.edu

1 Comparison of Computational Runtimes

The six different selection strategies are compared in terms of running time on the shortest (in terms of number of amino acids) and longest protein target from each of the three categories (easy, medium, and hard). Figure 1 shows the running times in a log-scale plot, so as to accommodate trivial strategies, such as Cluster-Random, and the most computationally-expensive strategy, Cluster-Size. The results show that the basin-based selection strategies are more efficient than Cluster-Size.

2 Impact of Energy-based Filtering on Selection Strategies

One can consider *a-priori* filtering of computed conformations prior to cluster- or basin-based selection, particularly as a strategy to reduce data size and thus computational time. Two opposite filtering strategies can be considered. One removes the x% lowest-energy conformations, whereas the other removes the x% highest-energy conformations.

Figure 2 shows the basins obtained when removing the 10% lowest-energy conformations from three representative test cases (one for each of the easy, medium, and hard categories described in the main article). As Figure 2 demonstrates, the removal of the lowest-energy conformations results in an explosion of basins with very low purity, causing the Pareto-based selection strategies to select low-purity basins. This is expected. Removing the lowest-energy conformations drastically changes the structure of the underlying landscape, as removing the lowest energies removes focal energies. Hence, such filtering results in many spurious basins on the deformed landscape.

The strategy of removing high-energy conformations (in other words, retaining lowest-energy conformations), on the other hand, preserves enough of the structure of the landscape, as shown in Figures 3 and 4. Figure 3 shows the basins (marking those selected by the Pareto-based strategies) obtained when the 50% highest-energy conformations are removed, whereas Figure 4 does so

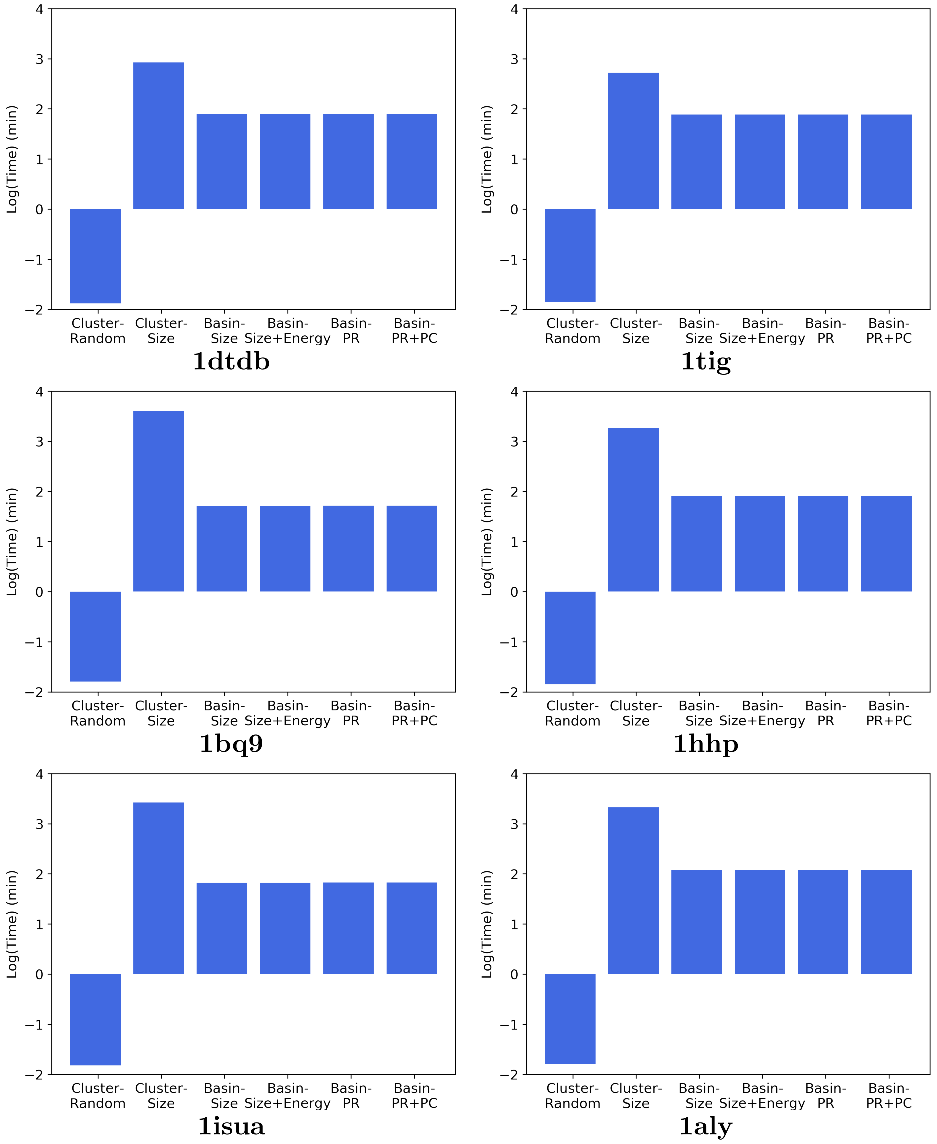

Figure 1: The six selection strategies are compared in terms of their running times in a log-scale on the shortest and longest target from the easy, medium, and hard categories.

after removing the 90% highest-energy conformations. As expected, removing higher-energy conformations does not drastically change the structure of the landscape and preserves enough basins for the basin-based selection strategies to hone in on high-purity ones.

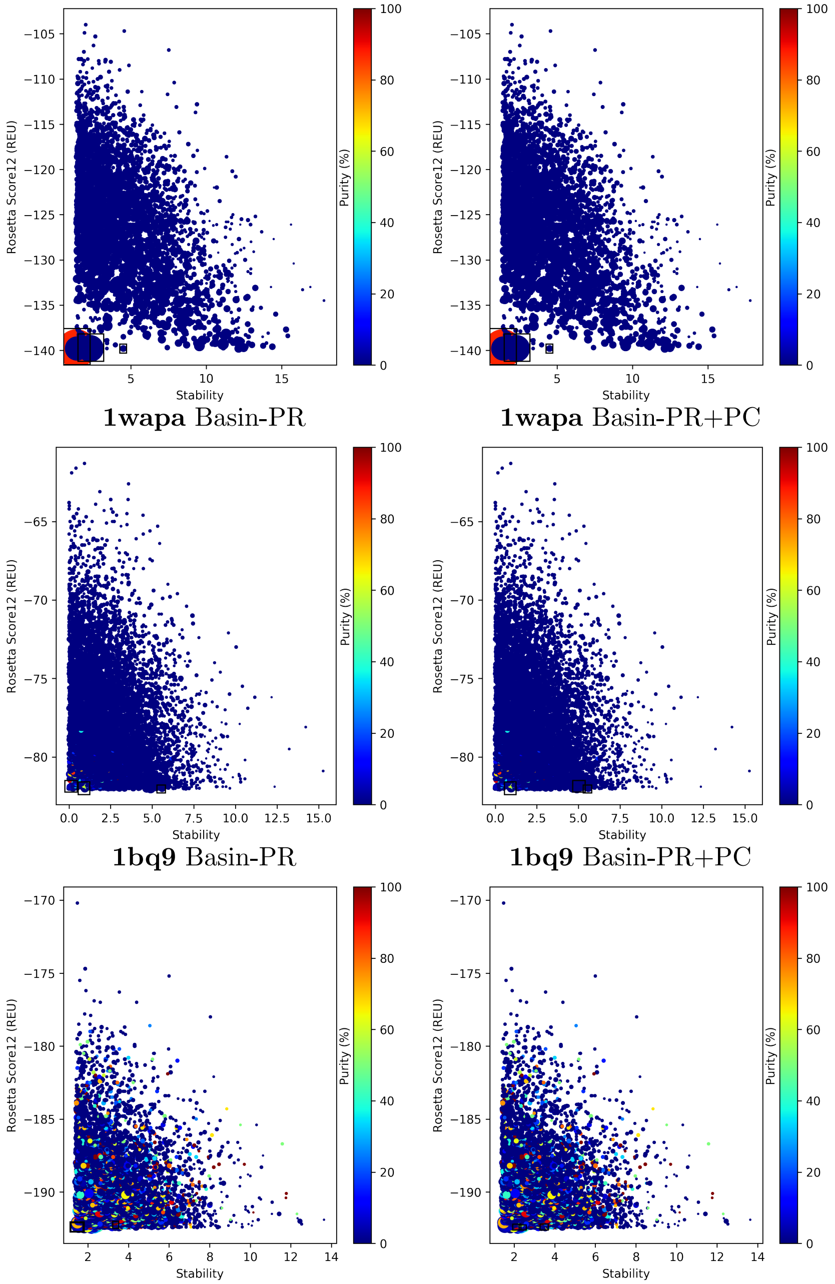

Figure 2: Visualization of basins extracted from the energy landscapes probed for an easy (PDB entry 1wapa), medium (1bq9), and hard target (2ezk) after removing the 10% lowest-energy conformations on each target. The color-coding scheme varies from blue (low purity) to red (high purity). The size of each disk respects the size of the corresponding basin. Top three basins selected by Basin-PR (left panel) and Basin-PR+PC (right panel) are indicated by encapsulating corresponding disks in rectangles.

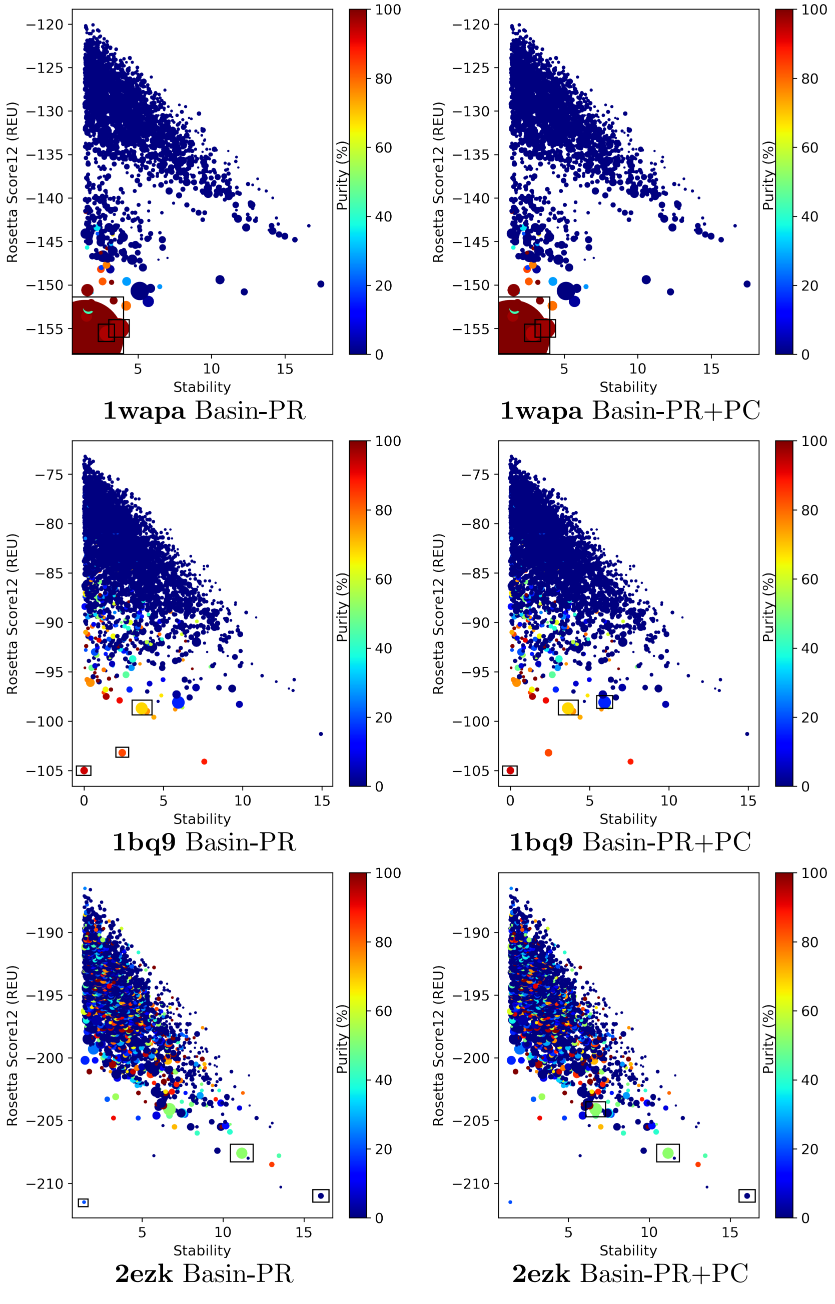

Figure 3: Visualization of basins extracted from the energy landscapes probed for an easy (PDB entry 1wapa), medium (1bq9), and hard target (2ezk) after retaining only the 50% lowest-energy conformations on each target. The color-coding scheme varies from blue (low purity) to red (high purity). The size of each disk respects the size of the corresponding basin. Top three basins selected by Basin-PR (left panel) and Basin-PR+PC (right panel) are indicated by encapsulating corresponding disks in rectangles.

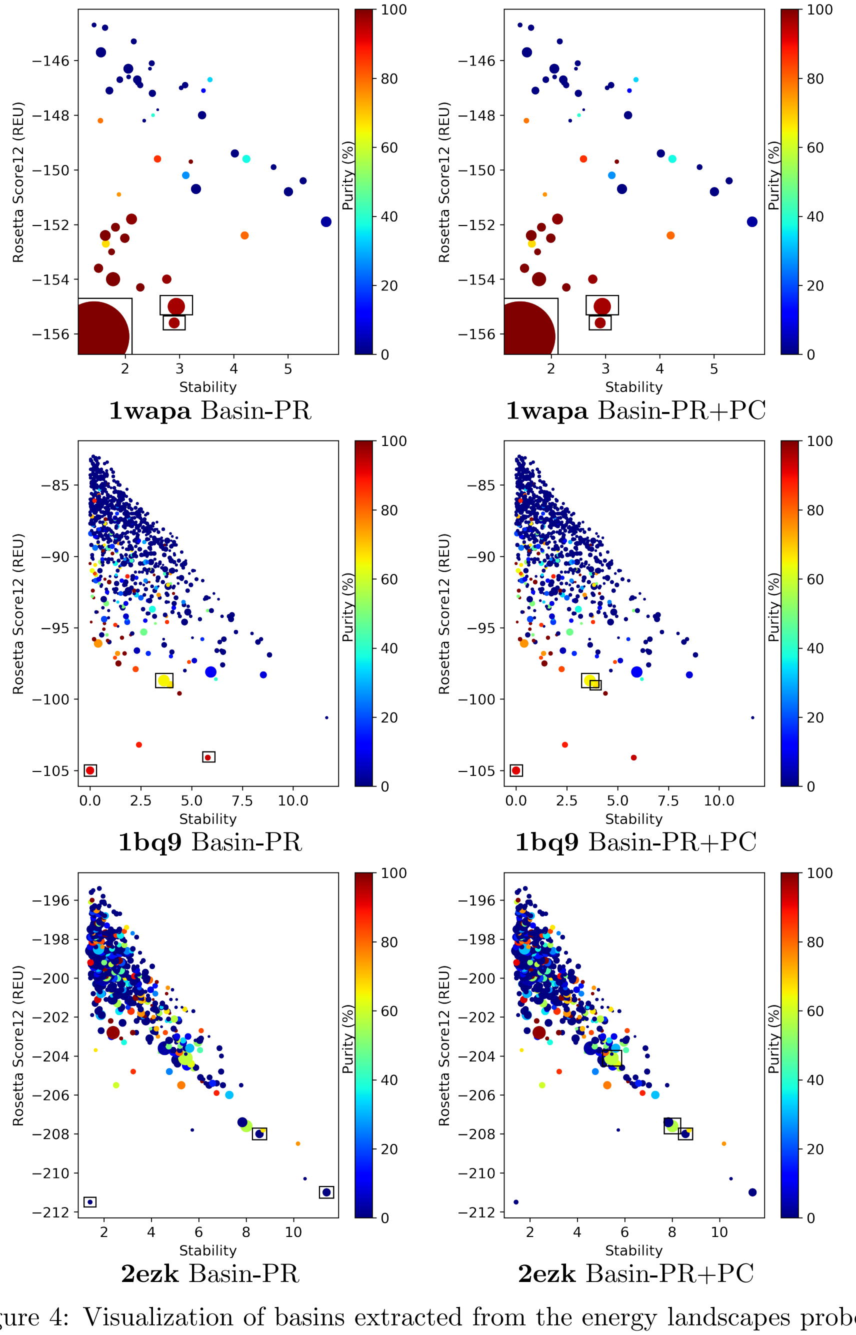

Figure 4: Visualization of basins extracted from the energy landscapes probed for an easy (PDB entry 1wapa), medium (1bq9), and hard target (2ezk) after retaining only the 10% lowest-energy conformations on each target. The color-coding scheme varies from blue (low purity) to red (high purity). The size of each disk respects the size of the corresponding basin. Top three basins selected by Basin-PR (left panel) and Basin-PR+PC (right panel) are indicated by encapsulating corresponding disks in rectangles.

3 Impact of Distance Threshold on Selection Strategies

The impact of different values of the `dist_thresh` parameter that determines which conformations are native (based on their lRMSD distance in `dist_thresh` to an experimentally-known native conformation) on the metrics n and p is now analyzed. The analysis is limited to the top cluster or basin selected and compares Cluster-Size, Basin-Size+Energy, and Basin-PR+PC. The plots below group the results on the different protein targets based on the three categories (easy, medium, and hard), as the ranges for `dist_thresh` are different for the three categories.

Figure 5 shows the impact on n (left panel) and p (right panel) on Cluster-Size and Basin-Size+Energy as `dist_thresh` is varied, and Figure 6 does so for Cluster-Size and Basin-PR+PC. Similar observations can be drawn from these two comparisons. As `dist_thresh` increases, n decreases and p increases. This is expected, as there is a scarcity of conformations sufficiently near to the known *native* conformation (e.g., < 1Å) in the dataset, especially for the targets in the medium and hard categories. Therefore, allowing larger distances from the known native conformation (i.e., larger values of `dist_thresh`) for a particular conformation to be deemed *native* increases the number of native conformations in a selection, which is reflected in higher purity.

For example, consider the target with known native conformation under PDB entry 2h5nd (bottom row of Figure 5). There are hardly any decoys closer than 6Å to the known native conformation. As a result, when `dist_thresh` is set to 6Å, the ratio (n) of the very few deemed-native conformations in the top basin selected by Basin-Size+Energy to the total number of native conformations (which is also very small) in the dataset results in a noticeable percentage, thus a high value of n. As `dist_thresh` increases, this percentage drops quickly, since the limited number of native conformations in a cluster or basin is now compared with a higher number of native conformations in the decoy dataset. On the other hand, as the the number of native conformations in a cluster or basin increases (with the increased value of `dist_thresh`), the ratio of that number to the size of the group/basin also increases, resulting in higher p.

On the easy targets (top row in Figure 5), varying `dist_thresh` does not significantly impact purity; with the exception of the target with known native conformation under PDB entry 1ail, satisfactory purity (around 85% at approximately 1.5Å) is achieved on all the easy targets. This behavior is expected, as there are a lot of decoys in the easy targets that are closer than 2Å to the known native conformation. The medium and hard targets show varying growth of p in response to varying `dist_thresh`. This is particularly the case for the medium targets, where most decoys are far away from the known native conformation. In the case of the target with known native conformation under PDB entry 1hz6a, good purity is achieved fairly quickly, whereas in the case of the target with known native conformations under PDB entry 1hhp, good purity is obtained only when `dist_thresh` becomes large.

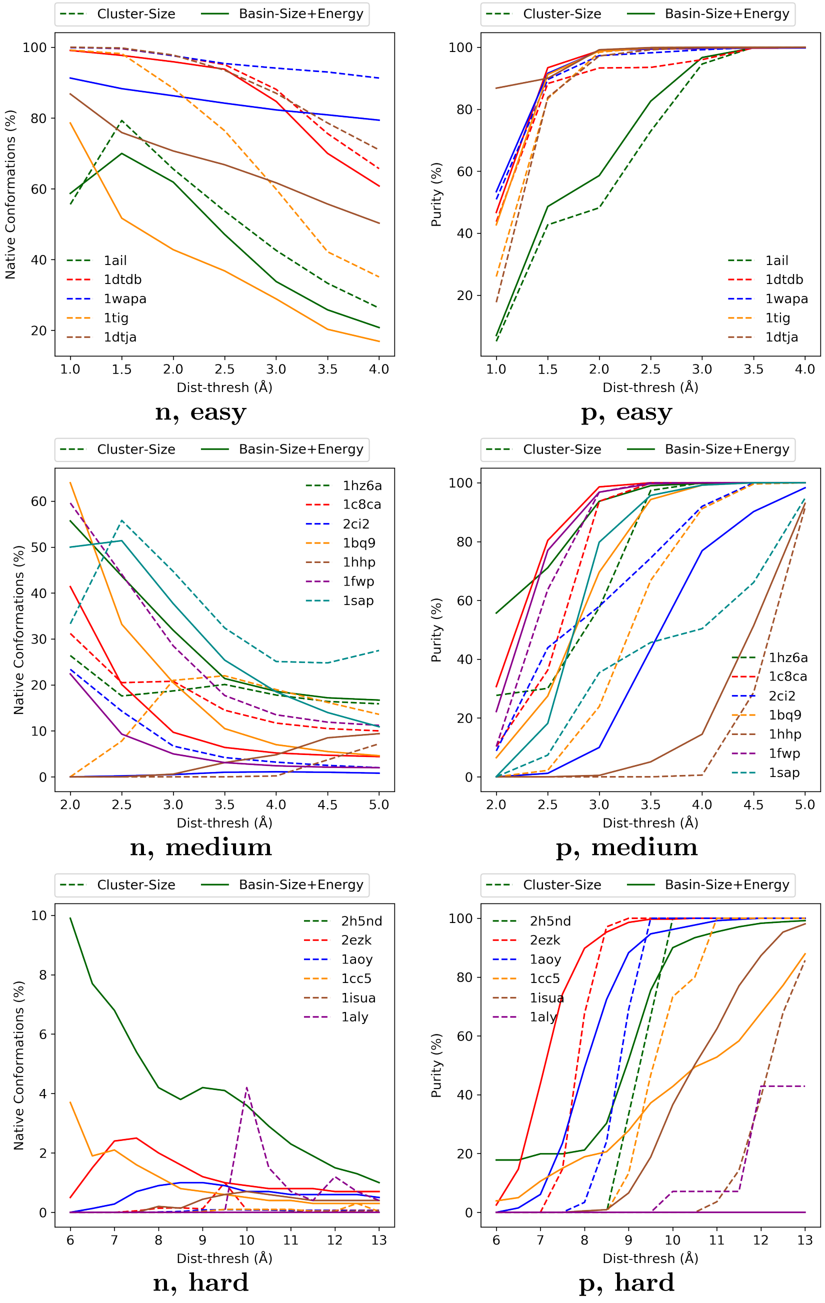

Figure 5: Impact on n (left panel) and p (right panel) on Cluster-Size and Basin-Size+Energy as `dist_thresh` is varied.

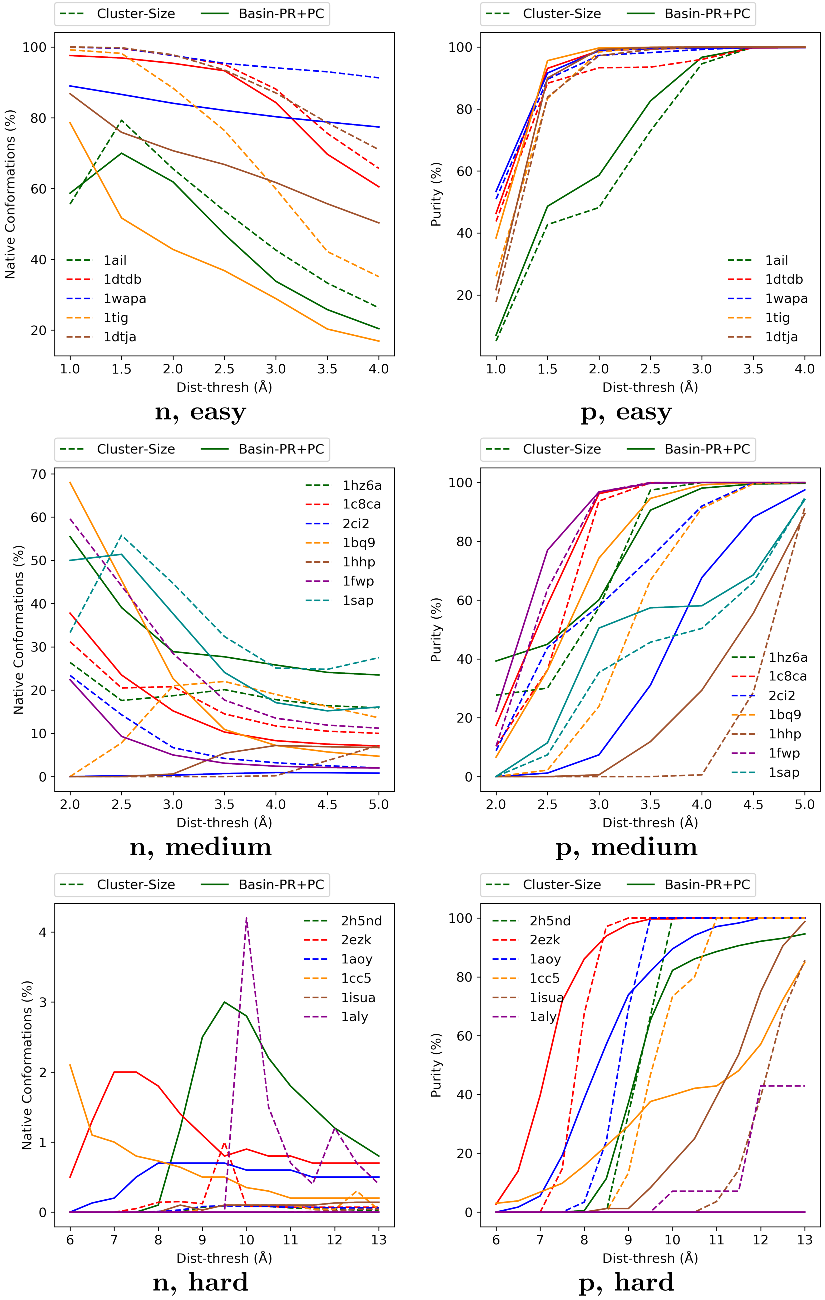

Figure 6: Impact on n (left panel) and p (right panel) on Cluster-Size and Basin-PR+PC as `dist_thresh` is varied.

Finally, in almost all targets (easy, medium, and hard), despite yielding a higher percentage of native conformations in the top cluster, Cluster-Size is outperformed by Basin-Size+Energy and Basin-PR+PC in terms of purity for varying `dist_thresh` values.

In summary, Figures 5-6 show that n decreases and p increases as `dist_thresh` increases. This implies that an evaluation of the performance of the selection strategies would yield comparatively-similar results at any specific `dist_thresh`. In the evaluation presented in the paper, we set `dist_thresh` so as to have the largest group selected by at least one selection strategy not be devoid of native conformations.
